# N-back task revisited: Comparing the neural correlates of updating and interference control

**DOI:** 10.1162/IMAG.a.1025

**Published:** 2025-11-24

**Authors:** Esmeralda Hidalgo-Lopez, Isabel Noachtar, Belinda Angela Pletzer

**Affiliations:** Department of Psychology & Centre for Cognitive Neuroscience, Paris Lodron University Salzburg, Salzburg, Austria; Department of Psychology, University of Michigan, Ann Arbor, MI, United States; Chronic Pain and Fatigue Research Center, University of Michigan Medical School, Ann Arbor, MI, United States

**Keywords:** working memory, fMRI, updating, interference control, functional connectivity

## Abstract

Working memory arises from the recruitment of multiple and multimodal specialized systems that support different executive functions, such as updating and interference control. This study revisits the n-back task, a classic working memory paradigm, directly comparing two trial types, targets and lures, to delineate the specific neural correlates of the updating and interference control within individual, and their modulation by different load conditions. This question is particularly relevant given evidence that balanced updating and interference control appear to be crucial for psychological resilience and mental health. We took an a priori approach by carefully selecting four different region-of-interest (ROI): bilateral middle frontal gyrus (MFG), inferior frontal gyrus (IFG), anterior insula, and caudate. A total of 159 healthy, right-handed young adult females (mean age = 23.33 ± 3.89 years) underwent fMRI while performing a verbal n-back task with two load levels and two stimulus types: targets and lures. Activation analyses were performed at both ROI and voxel-wise levels, and connectivity was examined through seed to voxel-wise analyses. Although updating and interference control showed an overlapping brain network, unique (and in some cases opposite) patterns arose for each process. These patterns include (i) stronger activation and differential connectivity of the MFG and caudate during targets compared with lures, (ii) increased cortical, but decreased subcortical activation with load during targets, while the opposite pattern emerged for lures, (iii) left lateralization for targets, but right lateralization during lures. This study uncovers the nuanced neural dynamics underlying updating and interference control during verbal working memory in females, underscoring the interplay between cortical and subcortical brain regions and their integration across cognitive demands.

## 1 Introduction

Working memory is an executive function that coordinates other higher cognitive processes to eventually guide our behavior through decision making (Miyake et al., 2000). Although its underlying mechanisms have been interpreted through multiple psychological frameworks (Baddeley & Hitch, 1974; Goldman-Rakic, 1992), lately, it has been described as an emergent property. As such, it arises from the recruitment of multiple and multimodal specialized systems that sustain the manipulation and maintenance of information (Postle, 2006). One of the key features that allows working memory integration is the so-called binding process between the incoming information and the mnemonic cognitive representation. Responding to stimulus features depending on internal rules requires a hierarchical integration of bottom-up and top-down processes. These integrative operations have been consistently reported to be modulated not only by the contextual features (i.e., task load), but also by individual characteristics (e.g., sex/gender or age) (Sander et al., 2011; Wang et al., 2019). Altogether, an efficient working memory requires the selection of external information, inhibition of irrelevant stimuli, and maintenance of the goal (Miyake et al., 2000).

The balance between these different processes has been linked to psychological resilience (Shariati et al., 2024) and mental health (Barkley, 2001; McTeague et al., 2016). Accordingly, a disbalance between working memory processes accompanies various mental health conditions, specifically stress-related disorders such as depression, anxiety, and post-traumatic stress disorder (Gohier et al., 2009; Rosa-Alcazar et al., 2021; Shaw et al., 2009). These disorders are twice as common in the female population and more likely to emerge during hormonal transition periods (Rubinow & Schmidt, 2006, 2019). Accumulating evidence suggests that adverse mood effects during hormonal transition periods are not only linked to a dysregulation between the brain networks relevant for mental health and resilience (Hidalgo-Lopez et al., 2023; Menon, 2011), but also to specific impairments in working memory (Hampson, 2018; Slyepchenko et al., 2017; Workman et al., 2012). Thus, understanding the extent to which different working memory processes recruit different cortical networks is crucial to consolidate the role of working memory balance in mental health, especially in the female population.

Recent approaches have begun to parcel specialized areas in the lateral prefrontal cortex (PFC) in relation to segregated aspects of working memory (Nee, 2021; Nee & D’Esposito, 2017). While the frontal pole and the most rostral portions of the middle frontal gyrus (MFG; Broadmann areas—BA9/10/46) are thought to support planning and top-down control, the posterior segments of the superior frontal gyrus (SFG) are associated with sensory-motor actions, whereas intermediate regions such as the inferior frontal gyrus (IFG; BA44/45) play a key role in binding processes and in flexibly guiding behavior according to contextual cues (Nee, 2021; Nee & D’Esposito, 2017). Accordingly, connectivity analyses show that these areas form specialized sub-networks with parietal areas (Nee, 2021).

Existing models also highlight the importance of subcortical loops for these processes. These models support that working memory relies on the coordination between the PFC and several subcortical areas like the striatum (McNab & Klingberg, 2008; Nee & D’Esposito, 2015; O’Reilly & Frank, 2006). Parallel cortico-striatal circuits allow for the planning, selection, retention, and response-related processes during the task (Alexander & Crutcher, 1990). For both sensorimotor and associative loops, prefrontal projections to caudate and putamen, and subsequent connections from these regions to premotor and motor cortices via the thalamus have been well characterized (Middleton & Strick, 2000; Parent & Hazrati, 1995). Specifically, selective updating has been modeled as a dynamic gating process via the caudate and thalamus, while information is maintained in the lateral PFC (O’Reilly & Frank, 2006). However, many of these processes have been studied in isolation and functional networks derived from mixed or male-dominated samples, so whether these patterns are similar in females remains largely unexplored.

It has also been proposed that information integration from these various cortical networks, as well as from subcortical areas during working memory processes, occurs in the anterior insular cortex, which is also heavily active during executive functions (Mencarelli et al., 2019; Wang et al., 2019). As a uniquely positioned hub, it integrates a wide range of top-down and bottom-up information (i.e., endogenous states, contextual features, cognitive processes, or motor action) (Cai et al., 2014; Gu et al., 2013; Sridharan et al., 2008) and serves as a switcher between large-scale brain networks depending on the contextual demands (Bunney et al., 2017; Flynn, 1999). While its role in executive control has been already described, whether its contribution differs across updating and interference control processes, and how these dynamics manifest exclusively in females, is less clear.

The n-back task has become a classic paradigm to study working memory and the factors that modulate its processes. By using different conditions, this task allows not only the assessment of continuous updating, that is, manipulating and retaining information, but also, the attentional control of interference (Burgess et al., 2011; Miyake et al., 2000). Updating and interference control can be studied through target and lure trials, respectively (Engle & Kane, 2003; Gray et al., 2003). In a stimulus sequence, target trials are stimuli identical to those presented exactly n trials before, and require an affirmative response, whereas lure trials are distractor stimuli that match a stimulus presented n+/-1 steps back and require a negative response. Although lures may elicit an affirmative response tendency, this tendency must be inhibited. The literature found activation for the updating process in dorsolateral and ventrolateral PFC, frontal pole, lateral and medial premotor cortex, dorsal cingulate, and bilateral and medial posterior parietal cortex, as well as subcortical structures (for metanalysis, see Owen et al., 2005). Regarding the interference control, IFG, insula, and anterior cingulate cortex (ACC) have been implicated in detection of conflict and interference resolution, with a crucial role of the right IFG during response execution (Botvinick et al., 2004; Nee et al., 2007). A greater top-down bias via the left IFG has been reported to facilitate the overcoming of interference during working memory processes (Burgess et al., 2011; Jonides & Nee, 2006), while other brain areas, such as the parietal lobes (BA39/40/7), have been shown to respond to the object identity of the n-back task in a load-dependent manner (Wang et al., 2019). Thus, although various studies have characterized the brain networks involved in updating and interference control, to the best of our knowledge, very few have compared directly the two processes using the same stimulus material and within the same participant. Specifically, while sex-specific working memory networks have been previously described (Hill et al., 2014), such comparisons have not yet been examined in an exclusively female sample. Our hypotheses and ROI selection were carefully based on the literature with sex-balanced samples; however, it remains largely unknown whether these mechanisms operate in the same way in females specifically. The n-back task provides the unique opportunity to directly compare both processes within a single task design and individual. Furthermore, the way in which interference control modulates brain activity and connectivity across different working memory loads remains insufficiently characterized.

In order to compare the specific neural correlates of the updating and interference control in working memory, and how these processes are modulated by cognitive load, we assessed the n-back task in a large sample of healthy adult females while controlling for inter-individual factors such as age and general intelligence. We took an *a priori* approach by carefully selecting four different region-of-interest (ROI) consistently implicated in working memory and load effects: the middle portion of MFG (BA9), the ventrolateral section of the IFG (BA44/45), anterior insula (BA13), and caudate, which also overlapped with networks functionally engaged in our task. Cognitive load was studied by contrasting a 0-back condition (purely attentional) with 2-back and 3-back conditions. Different activation and connectivity patterns were expected for updating (i.e., targets) and interference control (i.e., lures). We hypothesized that PFC activation during the updating would increase with load, while it would decrease during interference control (Chatham et al., 2011). Given the anterior insula’s role in binding and switching processes, we expected its activation and connectivity to increase with higher working memory load. Specifically for the caudate function, we hypothesized a differential activity between updating and interference control, and with increased activation during updating at higher loads (Chatham & Badre, 2015; O’Reilly & Frank, 2006). Finally, given the IFG’s well-established role in interference control, we expected it to be particularly sensitive to load during lure trials.

## 2 Materials and Methods

### 2.1 Participants

A total of 159 healthy right-handed young adult females participated in two different studies (n_1_ = 43, n_2_ = 116) with identical cognitive paradigms, and were included in the final analysis. Age (mean = 23.33, SD = 3.89) ranged from 18 to 36 years and was assessed as a continuous variable. Participants were recruited via flyers at the University of Salzburg and via online advertisements. Main exclusionary criteria were neurological, psychiatric or endocrine disorders, medication intake, or brain abnormalities displayed on structural MRI. Participants underwent a pretest to train the tasks and assess general intelligence (mean = 109.76, SD = 8.78) (John & Raven, 2003) (Advanced Progressive Matrices, Screening).

### 2.2 Ethics statement

Experiments were approved by the University of Salzburg’s ethics committee and were conducted in accordance with the Declaration of Helsinki. All participants gave their informed written consent to participate in the study.

### 2.3 Cognitive paradigm

Stimulus presentation and acquisition of behavioral data were done with Presentation® (http://www.neurobs.com/) on a CRT monitor (1024 × 768-pixel resolution, 120 Hz refresh rate). To assess verbal working memory, the verbal n-back task was adapted from Jacobs and D’Esposito (2011) as described in Hidalgo-Lopez and Pletzer (2021). Briefly, the n-back task consisted of 12 blocks (4 blocks each 0-back, 2-back, or 3-back) of 20 uppercase letters, presented sequentially for 1 s, followed by a 1-s inter-stimulus interval (fixation cross) and randomly interspaced by 5 null events (fixation cross). In each block, the participants needed to identify targets by responding “yes” and no-targets by responding “no”. Trials defined as targets were those with the letter “X” (0-back) or letters that had been presented 2 or 3 trials before (for 2- and 3-back, respectively). In the 2- and 3-back, trials defined as lures were those letters that match a stimulus presented n+/-1 trials earlier. In 2- and 3-back blocks, proportions were 20% targets, 65% non-lures, and 15% lures. In 0-back, proportions were 20% targets and 80% non-lures. All sequences were pseudo-randomized. Each block was preceded by 10 s and followed by 20 s of fixation cross. By using a within-subject mixed block/event-related design with adequate fixation periods, jittering, and within-subject contrasts, we ensured reliable estimation of both sustained and transient blood oxygenation level-dependent (BOLD) responses with fewer event trials per condition than purely event-related designs (Petersen & Dubis, 2012; Visscher et al., 2003).

### 2.4 fMRI data acquisition

For the two studies, functional scans lasted for 18 min and were obtained with a T2*-weighted gradient echo planar (EPI) sequence sensitive to BOLD contrast (whole brain coverage, descending, interleaved, TE  =  30 ms, TR  =  2250 ms, flip angle 70°, slice thickness 3.0 mm, matrix 192 × 192, FOV 192 mm), 36 transversal slices were taken oriented parallel to the AC-PC line. For the first study, functional and high-resolution structural images were acquired on a Siemens Magnetom TIM Trio 3 Tesla scanner following a field map acquisition. For the most recent study, images were acquired in the same scanner, updated to the Prisma fit software, but maintaining every parameter. High-resolution structural images were acquired with a T1-weighted sagittal 3D MPRAGE sequence (TE = 2.91 ms, TR = 2.30 ms, TI delay = 900 ms, flip angle 9°, slice thickness 1.00 mm, FOV 256 mm, voxel size 1.0 x 1.0 x 1.0 mm, 176 sagittal slices).

### 2.5 Preprocessing of fMRI data

The first six images of each session were discarded and the remaining scans were analyzed using standard procedures and templates from Statistical Parametric Mapping (SPM12, https://www.fil.ion.ucl.ac.uk/spm/software/spm12). These included the slice timing, co-registration of functional to structural images, segmentation of structural images using the computational anatomy toolbox for SPM (CAT12), and normalization of functional images using the parameters obtained by CAT12. Finally, the data were resampled to isotropic 3 × 3 × 3 mm voxels and smoothed with a Gaussian kernel of 6 mm. Additionally, as a first step, images were despiked using 3D-despiking as implemented in AFNI (afni.nimh.nih.gov). A biophysically based model (Functional Image Artefact Correction Heuristic, FIACH) (Tierney et al., 2016) was applied to identify and correct for non-physiological noise. Six regressors of physiological noise were extracted via principal components analyses from a time series signal-to-noise ratio (TSNR) map.

### 2.6 fMRI data analyses

At a first level, we modeled one regressor per trial type (0-back: non-lures, targets; 2-back and 3-back: non-lures, lures, targets). All regressors were obtained by convolving the duration of the event with the canonical hemodynamic response function implemented in SPM12. A high pass filter cut-off was set at 128 s and autocorrelation correction was performed using an autoregressive AR(1) model (Friston et al., 2002). To assess the BOLD response for targets and lures, the following contrasts were defined: (i) 2-back targets >0-back targets, (ii) 3-back targets >0-back targets, (iii) 2-back lures >2-back non-lures, (iv) 3-back lures >3-back non-lures. To assess the differences between targets and lures, we further define contrasts for (v) 2/3-back targets >0-back targets, (vi) 2/3-back lures >2/3-back non-lures. Finally, to normalize for individual differences in baseline fluctuations, the contrasts were scaled Kalcher et al. (2013) by dividing the contrast image by the amplitude of low-frequency fluctuation (ALFF) map (Zang et al., 2007) using the Data Processing for Analysis of Brain Imaging (DPABI) toolbox (Yan et al., 2016) with a band-pass filter of 0.01–0.08 Hz.

**Fig. 1.**
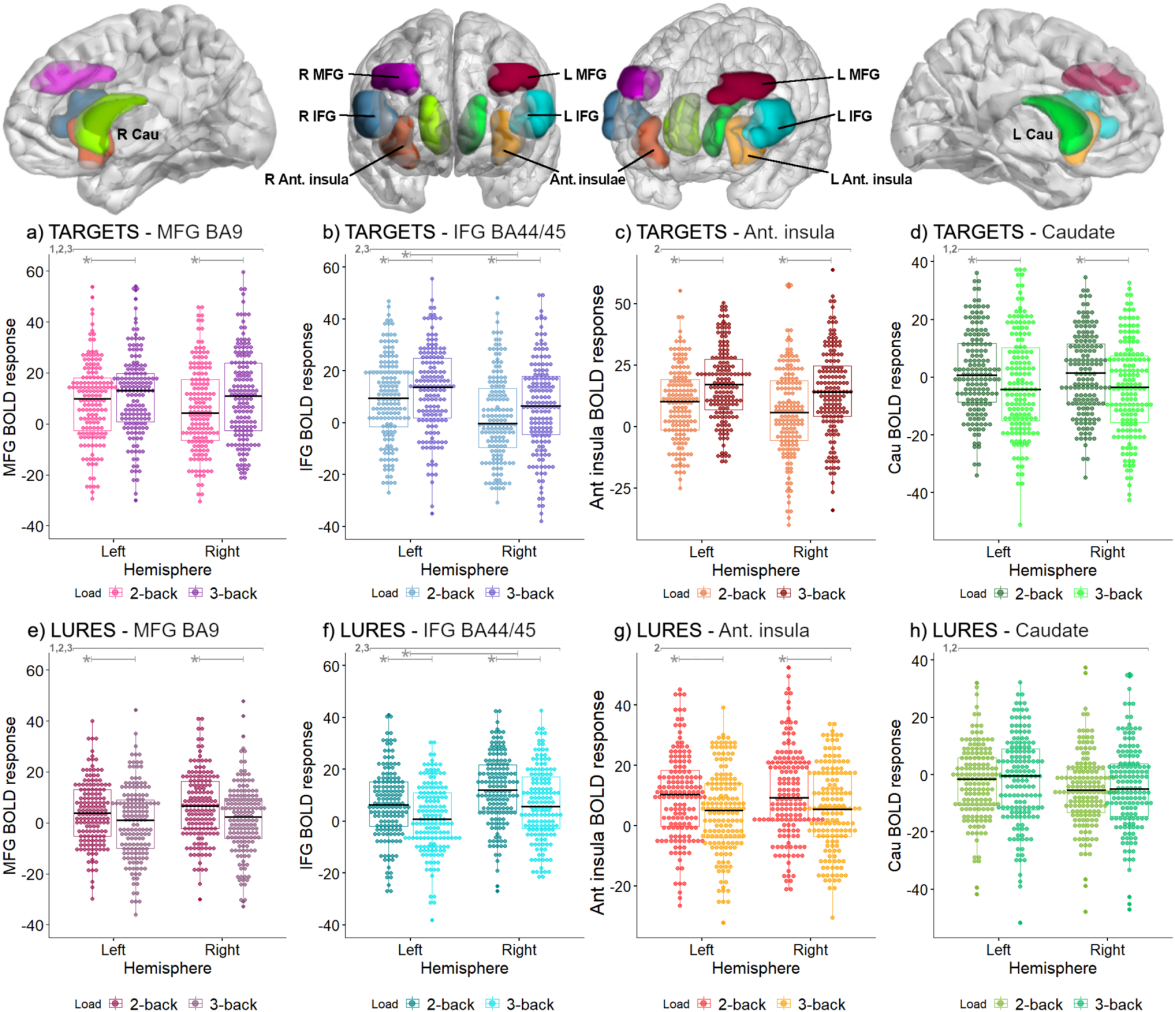
BOLD response of the regions of interest (ROIs) during targets (a–d) and lures (e–h), for each condition and hemisphere. ROIs were composed of (a,e) bilateral middle frontal gyri (MFG; Brodmann area 9), (b,f) inferior frontal gyri (IFG; Brodmann areas 44/45), (c,g) anterior insulae, and (d,h) caudate. BOLD response is expressed by the difference between 2- and 3-back versus 0-back for targets, and the difference between 2-back lures versus 2-back non-lures, and 3-back lures versus 3-back non-lures for lures. MFG: middle frontal gyrus; IFG: inferior frontal gyrus; Ant: anterior. In gray lines *p_FDR_ < 0.05; 1 indicates trial type significant differences, 2 indicates significant interaction of trial type by load, 3 indicates significant interaction of trial type by hemisphere; see Table 2 for specific p-values.

At a second level, we first focused on a region-of-interest-based (ROI) analysis and then examined the voxel-wise level. For ROI analyses, first, each of the following first-level contrasts were entered into a one-sample t-tests separately for targets and lures: 2-back targets>0-back targets, and 3-back targets>0-back targets for targets; and 2-back lures>2-back non-lures, 3-back lures>3-back non-lures for lures. Then, eigenvalues were extracted from bilateral ROIs: (i) middle frontal gyrus (MFG) section corresponding to BA9, (ii) inferior frontal gyrus (IFG) section corresponding to BA44 and BA45, (iii) anterior insula, and (iv) caudate. We used anatomically pre-defined ROIs to maximize generalizability and comparability with prior studies, encompassing peak coordinates from meta-analyses (e.g., Owen et al., 2005) and overlapping regions showing task-related effects in our voxel-wise analyses (compare with Supplementary Table S1), thereby minimizing peak selection bias while ensuring functionally relevant signal. ROIs were built using the ImCalc option in SPM12 and the MarsBaR toolbox (Brett et al., 2022). (i) and (ii) were defined as the intersection of (i) MFG and (ii) IFG (AAL atlas; Tzourio-Mazoyer et al., 2002) with respective Brodmann areas as implemented in the Wake Forest University (WFU) Pickatlas toolbox (Maldjian et al., 2003). Anterior insula (iii) was defined as the most anterior section of AAL atlas-defined insula, and caudate (iv) was also AAL atlas defined (Tzourio-Mazoyer et al., 2002). ROIs are depicted in Figure 1 with the BrainNet Viewer (Xia et al., 2013) (http://www.nitrc.org/projects/bnv/). Eigenvalues from these ROIs were extracted without threshold and entered into linear-mixed effects models as dependent variables, accounting for multiple comparisons correction (compare *Statistical analysis* section below).

Second level voxel-wise activation analyses were assessed by entering each of the following first level contrasts into two different flexible factorial designs: 2-back targets>0-back targets, and 3-back targets>0-back targets for targets; and 2-back lures>2-back non-lures, 3-back lures>3-back non-lures for lures, and modeling the factors subject and load. Age, general intelligence, and scanner type were added as covariates. Contrasts comparing 3-back versus 2-back within subjects were defined as described by Gläscher and Gitelman (2008). We further explored differences in the voxel-wise activation during targets versus lures including contrasts for 2/3-back targets>0-back targets, and 2/3-back lures>2/3-back non-lures into a flexible factorial design with subject, and trial type as factors. Load and trial type interaction was not explored in the voxel-wise analyses (only in the ROI analyses, see *Statistical analysis* section). Age, general intelligence, and scanner type were added as covariates. Contrasts comparing targets versus lures within the same participant, were defined as described by Gläscher and Gitelman (2008).

For the voxel-wise analyses, results were masked with an SPM gray matter template and thresholds were set as follows: k = 30 voxels extent, primary threshold: p < 0.001 uncorrected, secondary threshold: p < 0.05 cluster-level FWE corrected (p_FWE_). Voxel-wise analyses are briefly reported in the main text and further detailed in the Supplementary Material.

### 2.7 Seed-based connectivity analysis

We investigated the relationship between working memory load and seed-to-voxel connectivity of pre-defined ROIs using the CONN-toolbox standard procedures and templates (Whitfield-Gabrieli & Nieto-Castanon, 2012). Seed-to-voxel connectivity maps from these ROIs were estimated separately for targets and lures. In CONN, connectivity maps are computed as the Fisher-transformed bivariate correlation coefficients between the mean BOLD time series from a given ROI (seed) and the BOLD time series at each voxel in the brain (Whitfield-Gabrieli & Nieto-Castañón, 2012). These maps reflect both local homogeneity within the seed region and long-range functional connectivity with other brain areas. Potential confounding effects were addressed using CONN’s default denoising pipeline, which implements an anatomical component-based noise correction procedure (aCompCor; Behzadi et al., 2007). Six movement parameters, as well as five white matter and cerebrospinal fluid components, were used as regressors during the denoising step. In order to ensure that the condition-specific connectivity measures were not confounded by simple task-related co-activation effects, we regressed out each task condition as previously recommended (Nieto-Castanon, 2020). A simultaneous band-pass filter of 0.008–inf Hz was applied for the denoising step as recommended in Hallquist et al. (2013). Condition-specific connectivity maps were generated separately for each trial type (targets and lures) and load condition (0-, 2-, and 3-back) using the corresponding condition-specific time series from the denoised data. Load effects within each trial type were examined using first-level contrasts: 2-back targets>0-back targets, and 3-back targets>0-back targets for targets; and 2-back lures>2-back non-lures, 3-back lures>3-back non-lures for lures, and including subject as a factor. These contrasts isolated connectivity changes associated with higher working memory load for each trial type within each participant. Between-condition differences (targets vs. lures) were examined at the second level using a flexible factorial design in SPM12 with factors for subject and trial type, in the same way as for the activation analyses. Furthermore, associations between connectivity patterns and performance were addressed by adding RT increases and accuracy decreases in the 2- and 3-back condition as continuous regressors in separate flexible factorial models for target and lure connectivity maps. Covariates and thresholds were set as in the second level activation analyses.

### 2.8 Statistical analysis

Statistical analyses were carried out in R 3.6.2 using the lmer function of *lme4* package (Bates et al., 2015). Graphics are depicted using the *ggplot2* package (Wickham, 2009). Since targets and lures assess functionally different processes, they were analyzed separately (Burgess & Braver, 2010; Hidalgo-Lopez & Pletzer, 2021). In a first step, behavioral variables (*RT* and *accuracy*) were operationalized to match the brain activation and connectivity analyses. In order to do so, the performance of the control condition (0-back for targets; non-lures for lures) was subtracted from the condition of interest. For behavioral analyses, models included participant number (*VP*) as random factor, *IQ* and *study group* as covariates, and load and age as effect of interest (e.g., *RT*~1|*VP*+load*trial type+*age+IQ*+*group*). Outliers for each trial type were defined as those surpassing a 3SD threshold for either *accuracy* or *RT*. A total of seven participants for the targets and two participants for the lures were removed from subsequent analyses. For the models including ROI activation, we additionally included hemisphere as covariate (e.g., *BOLD_MFG_*~1|*VP*+load*trial type*hemisphere+*age*+*IQ*+*group*). We finally examined whether the ROIs activity could predict performance while controlling for load and hemisphere: *RT*~1|*VP*+BOLD response*load*hemisphere+*group*. In all models, both the dependent and continuous independent variables were scaled so that the coefficients b represent standardized effect sizes based on standard deviations (comparable with Cohen’s d). Standard error of this coefficient is indicated by SEb. Non-significant interactions were backward eliminated, to facilitate meaningful interpretation of the main effects. For the models including brain activation and connectivity, we accounted for multiple testing further FDR correcting the p-values for the four bilateral ROIs with *p.adjust* function in *stats* package (R Team, 2019).

## 3 Results

Table 1 summarizes the descriptive statistics and Table 2 the statistical parameters (F-values) of the behavioral and ROI-based analyses. Effect sizes are reported in the text.

**Table 1.** Descriptive statistics (mean ± SD) for behavior and ROI-based activation.

	Left hemisphere				Right hemisphere			
	Targets		Lures		Targets		Lures	
	2-back	3-back	2-back	3-back	2-back	3-back	2-back	3-back
Accuracy	-6.99 ± 14.72	-23.00 ± 16.31	-23.51 ± 17.06	-33.76 ± 16.54				
RT	114.65 ± 80.64	185.77 ± 102.37	130.88 ± 95.44	128.86 ± 129.81				
MFG	8.67 ± 15.76	11.80 ± 16.61	4.01 ± 12.36	0.27 ± 14.45	5.80 ± 16.76	10.95 ± 17.62	6.62 ± 13.20	1.78 ± 13.42
IFG	9.19 ± 16.34	13.08 ± 17.01	6.62 ± 13.43	1.32 ± 13.03	1.78 ± 17.00	6.35 ± 17.77	12.23 ± 13.40	6.58 ± 14.00
Ant insula	9.30 ± 15.19	17.51 ± 15.17	9.83 ± 14.23	4.89 ± 13.92	5.86 ± 17.77	14.25 ± 17.49	10.49 ± 15.04	5.52 ± 13.92
Caudate	1.69 ± 14.39	-2.02 ± 17.71	-2.56 ± 12.61	-1.71 ± 15.36	1.13 ± 14.11	-3.89 ± 16.41	-5.33 ± 12.61	-4.36 ± 14.59

Values show the difference between the control condition (0-back for targets; non-lures for lures) and the condition of interest. RT: reaction time; MFG: middle frontal gyrus; IFG: inferior frontal gyrus; Ant: anterior.

**Table 2.** Statistical parameters (F-values) for evaluating differences in behavior and brain activation across load, trial type, hemisphere, and age during the n-back paradigm.

	Age	Load	Trial Type	Load by Trial Type	Hemisphere	Load by Hemisphere	Trial Type by Hemisphere	Load by Trial Type by Hemisphere
Accuracy	4.63^*^	104.18^***^	107.86^***^	8.79^**^				
RT	12.91^**^	37.12^***^	1.9	20.80^***^				
MFG	11.17^**^	4.49^*^	8.91^*^	10.52^**^	3.11	0.65	6.12^*^	0.83
IFG	2.46	5.73^*^	2.99	16.61^***^	22.57^***^	0.13	34.47^***^	0.15
Ant insula	2.42	31.67^***^	0.29	40.08^***^	4.78	<0.01	3.46~	<0.01
Caudate	6.52^*^	5.87^*^	8.30^*^	4.29^*^	0.28	0.3	0.91	0.18

*p_FDR_ < 0.05, **p_FDR_ < 0.01, ***p_FDR_ < 0.001. RT: reaction time; MFG: middle frontal gyrus; IFG: inferior frontal gyrus; Ant: anterior.

### 3.1 Behavioral results

Accuracy was significantly lower for lures than for targets (b = -0.94, SEb = 0.09) with no differences between trial types in RT (b = 0.14, SEb = 0.10). Accuracy decreased and RT increased with load (Acc: b = -0.93, SE_b_ = 0.09; RT: b = 0.63, SE_b_ = 0.10) and age (Acc: b = -0.08, SE_b_ = 0.04; RT: b = 0.16, SE_b_ = 0.04). However, the interactive effect between load and trial type indicated that associations with load were stronger for targets than for lures (Acc: b = 0.38, SE_b_ = 0.13; RT: b = -0.67, SE_b_ = 0.15; compare Tables 1 and 2).

### 3.2 ROI analyses

Like for behavior, the associations of the BOLD response to load were strongly moderated by trial type (compare Tables 1 and 2; Figure 1).

BOLD response was significantly stronger for targets than for lures in the MFG (b = -0.30, SEb = 0.10) and caudate (b = -0.29, SEb = 0.10), but not in the IFG (b = -0.17, SEb = 0.10) and anterior insula (b = 0.05, SEb = 0.10).

Irrespective of hemisphere, BOLD response in the cortical ROIs (MFG, IFG, and anterior insula) was positively related to load for targets (all b > 0.18, all SE_b_ < 0.09), but negatively for lures (all b < -0.27, all SE_b_ = 0.10). Conversely, BOLD response of the caudate decreased with load for targets (b = -0.24, SE_b_ = 0.07), but was not significantly related to load for lures (b = 0.05, SE_b_ = 0.10).

Lateralization was also moderated by trial type for the cortical ROIs. While activation was more left lateralized for targets (MFG: b = -0.16, SEb = 0.05; IFG: b = -0.43, SE_b_ = 0.08; anterior insula: b = -0.20, SE_b_ = 0.07), it was more right lateralized for lures (MFG: b = 0.20, SE_b_ = 0.10; IFG: b = 0.40, SE_b_ = 0.10). Age was negatively related to the BOLD response in the MFG (b = -0.13, SE_b_ = 0.04) and caudate (b = -0.11, SE_b_ = 0.04).

### 3.3 Voxel-wise activation

**Fig. 2.**
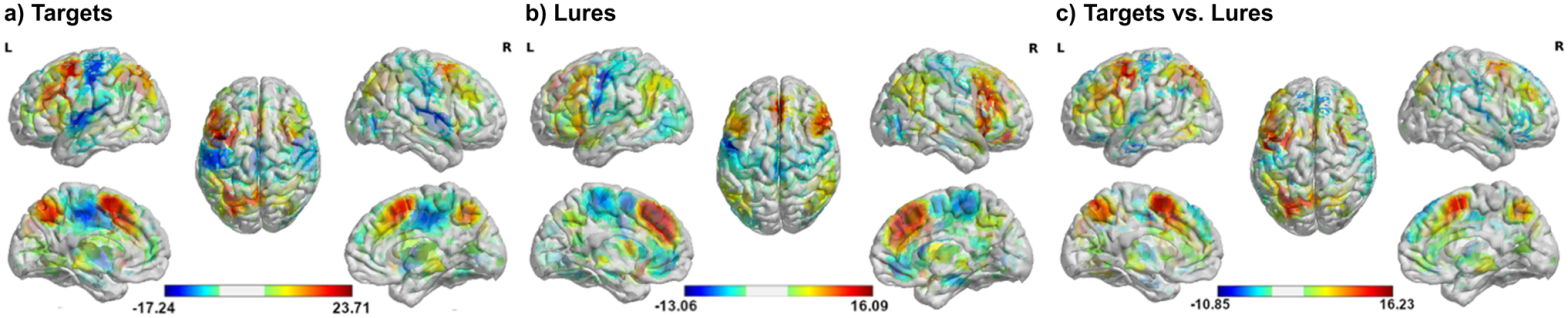
Voxel-wise task-related activation. (a) BOLD response during target trials compared with 0-back trials. (b) BOLD response during lure trials compared with non-lure trials. (c) Direct comparison of BOLD changes between target and lure trials. Warm colors (red/yellow) indicate increased BOLD response, while cool colors (blue) indicate decreased BOLD response. L = left hemisphere; R = right hemisphere. Color bars represent t-values.

In line with previous meta-analyses (Mencarelli et al., 2019; Owen et al., 2005), both target and lure trials increased the BOLD response of frontoparietal areas typically related to working memory (see Fig. 2a, b; Supplementary Table S1). In general, brain activation was more frontoparietal—left lateralized during targets while medial and temporal areas were more active during lures (see Fig. 2c; Supplementary Table S2).

### 3.4 Seed to voxel-wise connectivity analyses

#### 3.4.1 Trial type

**Fig. 3.**
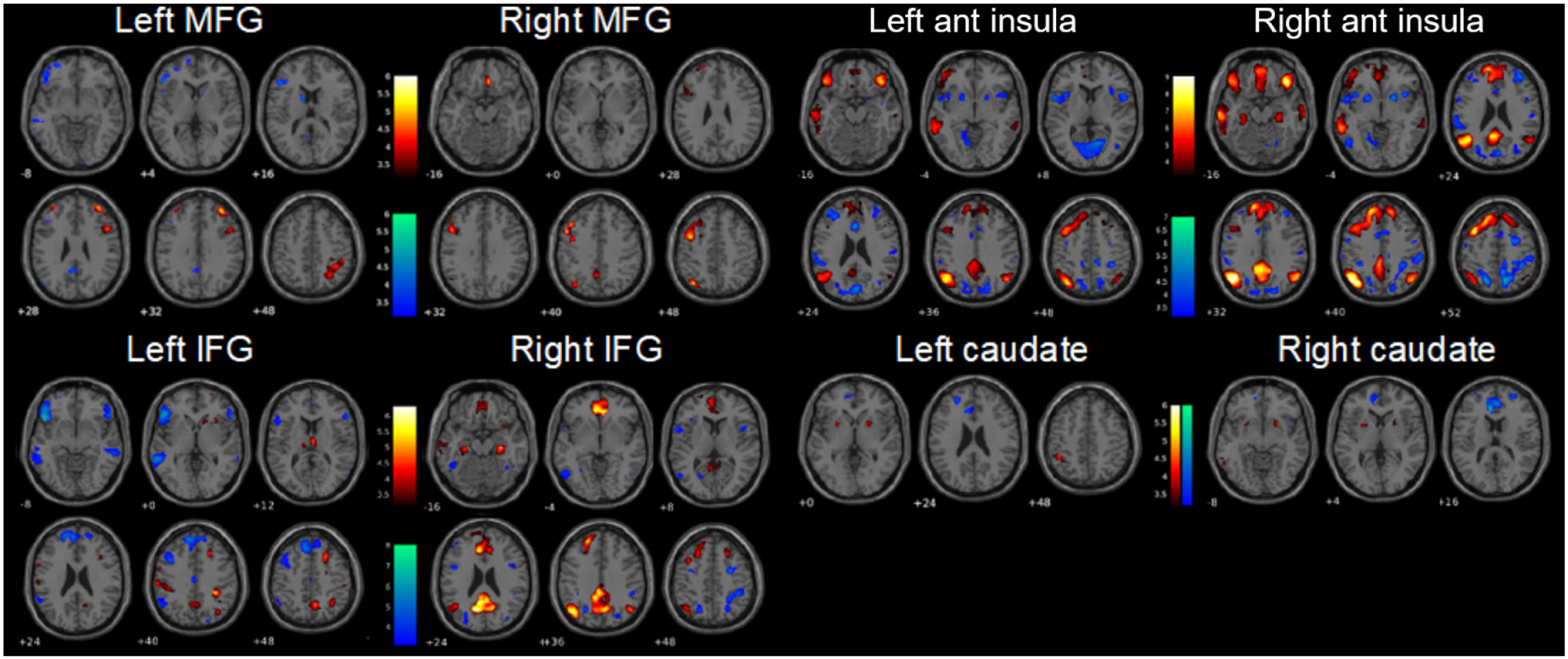
Connectivity maps for each ROI illustrating differential connectivity during targets relative to lures (targets > lures) and during lures relative to targets (lures > targets). Increased connectivity during 2/3-back targets compared with 2/3-back lures is displayed in warm colors, while increased connectivity during 2/3-back lures compared with 2/3-back targets is displayed in cold colors. MFG: middle frontal gyrus; IFG: inferior frontal gyrus; Ant: anterior.

Seed to voxel-wise connectivity patterns with the ROIs as seed for the target and lure trials, respectively, are described in the Supplementary Material (Figs. S4 and S5). Differences in the connectivity maps of each seed were also found between 2/3-back targets and 2/3-back lures (Fig. 3).

Stronger connectivity during targets compared with 0-back, while diminished during lures compared with non-lures was found between bilateral MFG with each other, and contralateral superior parietal lobule (SPL); between the left inferior frontal gyrus (IFG) and the right middle frontal gyrus (MFG) and inferior frontal gyrus (IFG), right superior parietal lobule (SPL), posterior cingulate cortex (PCC), left precentral and postcentral gyri, and right thalamus; between the right inferior frontal gyrus (IFG) and the posterior cingulate cortex (PCC), anterior cingulate cortex (ACC), bilateral parahippocampal and hippocampal regions, and bilateral angular gyri (AG); between bilateral anterior insula and bilateral AG, left MFG, bilateral orbital IFG, bilateral precuneus, and temporal areas; between right anterior insula and bilateral para/hippocampus; and between bilateral caudate and bilateral putamen.

On the contrary, lower connectivity strength during targets compared with 0-back, while increased during lures compared with non-lures was found between left MFG and left caudate, and left orbital IFG; between left IFG and bilateral inferior orbital gyrus, left MFG, bilateral medial SFG, left AG, and bilateral temporal areas; between right IFG and bilateral MFG, SPL, left opercular IFG, and right insular cortex; between bilateral anterior insula with each other, right SPL, bilateral MFG and putamen; between left anterior insula and ACC, bilateral frontal operculum, and cuneus; between right anterior insula, right SFG, and occipital areas; and between bilateral caudate and the ACC.

#### 3.4.2 Load

**Fig. 4.**
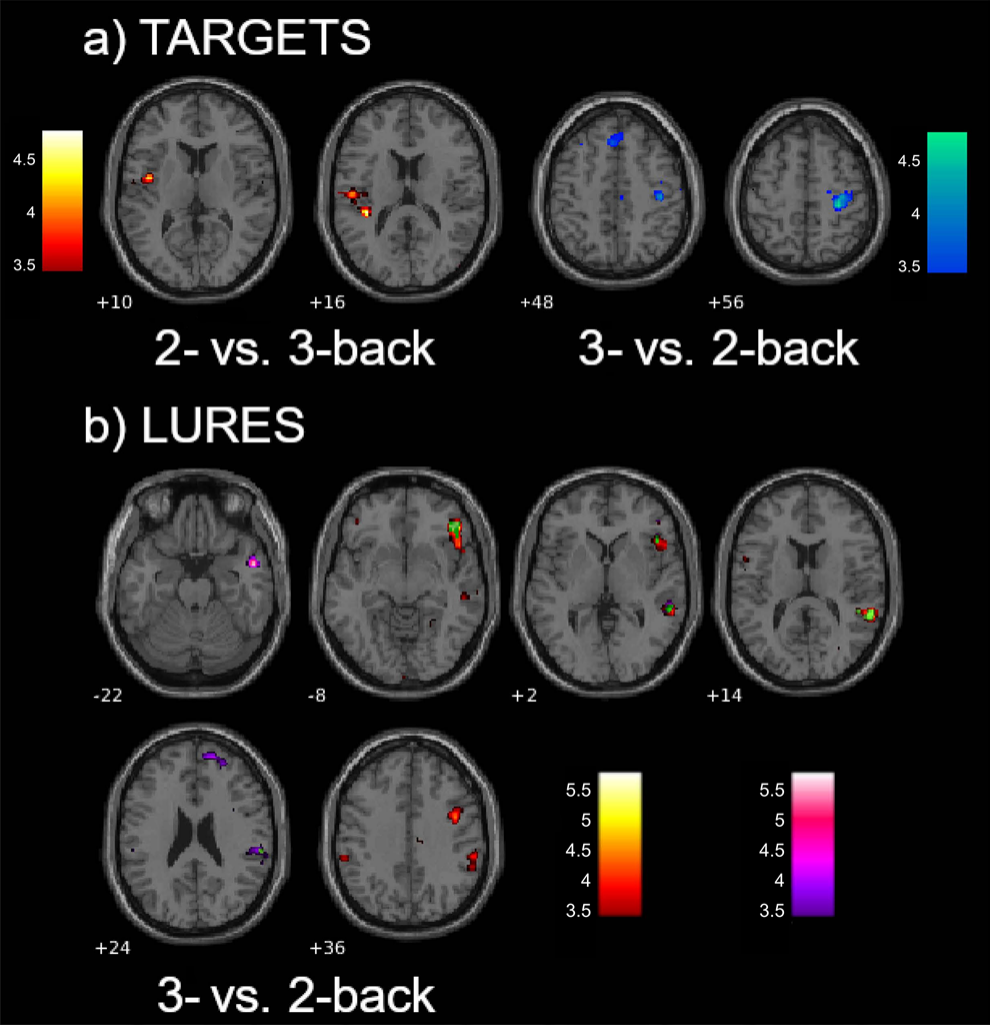
Association of load with the seed to voxel-wise connectivity during (a) targets and (b) lures. (a) During targets, connectivity decreased with load, between left inferior frontal gyrus and the left medial superior frontal gyrus, and between right anterior insula and right precentral/ postcentral gyrus (in cold colors); while connectivity increased between the right inferior frontal gyrus and left central operculum/insula (in red/orange). (b) During lures, connectivity increased with load between the bilateral anterior insula, right inferior frontal gyrus, and superior temporal gyrus (overlap showed in green); left anterior insula and right precentral gyrus and middle temporal gyrus (in red/orange); and right anterior insula and right anterior prefrontal cortex and superior temporal gyrus (in purple/pink). Color bars indicate t-values.

For targets, connectivity between the left IFG and the left medial superior frontal gyrus, and between the right anterior insula and right somatomotor cortices decreased during the 3-back compared with the 2-back. Connectivity between the right IFG and left central operculum/insula increased during the 3-back compared with the 2-back targets (see Table 3; Fig. 4a). For lures, higher load was related to increased connectivity between the bilateral anterior insulae and the right IFG, supramarginal gyrus (SMG), and middle/superior temporal gyri (MTG/STG), as well as between the left anterior insula and right precentral gyrus and between the right anterior insula and the right frontal pole (see Table 3; Fig. 4b).

**Table 3.** Association of load with the seed to voxel-wise connectivity.

**a)**	**TARGETS**										
	**2- vs. 3-back**					**3- vs. 2-back**					
	**cluster p_FWE_**	**Vx**	**T**	**x,y,z**	**Area**		**cluster p_FWE_**	**Vx**	**T**	**x,y,z**	**Area**
L IFG	0.019	35	4.03	-6 29 43	L mSFG	R IFG	0.017	35	4.48	-42 -4 10	L CO/Ins
R Ant insula	<0.001	887	4.39	33 -19 55	R PrePoCG							

**b)**	**LURES**										
	**3- vs. 2-back**					**3- vs. 2-back**					
	**cluster p_FWE_**	**Vx**	**T**	**x,y,z **	**Area**		**cluster p_FWE_**	**Vx**	**T**	**x,y,z**	**Area**
L Ant insula	<0.001	138	5.27	45 35 -8	R IFG	R Ant insula	0.02	34	3.97	45 35 -08	R IFG
	<0.01	61	4.05	60 -25 25	R SMG		<0.001	101	5.67	60 -43 13	R SMG
	<0.01	63	4.10	60 -43 4	R MTG		0.01	39	5.63	54 02 -20	R STG
	<0.01	42	4.28	42 2 37	R PreCG		0.01	37	4.55	18 56 25	R FrPole

(a) Areas that showed connectivity changes related to load during targets (compared with 0-back). (b) Areas that showed connectivity changes related to load during lures (compared with non-lures). R: right; L: left; IFG: inferior frontal gyrus; mSFG: medial superior frontal gyrus; PreCG: precentral gyrus; PoCG: postcentral gyrus; CO: central operculum; Ins: insula; STG: superior temporal gyrus; MTG: middle temporal gyrus; Ant: anterior; Fr: frontal.

#### 3.4.3 Age

Irrespective of the load, and during targets, the left MFG showed stronger connectivity with bilateral SMG (left: [-54, -31, 37], 69 voxels, T = 4.86, p_FWE_ < 0.001; right: [54, -37, 34], 40 voxels, T = 4.13, p_FWE_ < 0.01), the older the participant (Supplementary Material, Fig. S3).

### 3.5 Prediction of performance

For targets, better accuracy and faster reactions were related to higher BOLD response of the MFG (accuracy: b = 0.10, SE_b_ = 0.03, t_(614)_ = 3.10, p_FDR_ < 0.01; RT: b = -0.15, SE_b_ = 0.04, t_(617)_ = -3.89, p_FDR_ < 0.001) and caudate (accuracy: b = 0.12, SE_b_ = 0.03, t_(616)_ = 3.32, p_FDR_ < 0.01; RT: b = -0.10, SE_b_ = 0.04, t_(614)_ = -2.63, p_FDR_ = 0.01), but not the IFG (all b < 0.03 all t < 0.9, all p_FDR_ > 0.05). However, for the anterior insula, a higher BOLD response was related to slower reactions (b = 0.13, SE_b_ = 0.08, t_(615)_ = 3.07, p_FDR_ < 0.01). Higher accuracy was related to weaker connectivity between frontal ROIs and parietal areas. Specifically, accuracy was negatively related to connectivity between the left MFG and the left postcentral gyrus ([-33, -34, 52], 51 voxels, T = 4.56, p_FWE_ = 0.003), the right IFG and the left IPL ([-54, -52, 22], 41 voxels, T = 3.91, p_FWE_ = 0.013), as well as the left anterior insula and the left SMG ([-42, -34, 37], 31 voxels, T = 4.74, p_FWE_ = 0.046), and the left anterior insula and the right postcentral gyrus ([39, -28, 43], 33 voxels, T = 4.37, p_FWE_ = 0.036). In addition, target accuracy was negatively related to the strength of connections between the right frontal ROIs (right IFG–right MFG: [36, 14, 43], 55 voxels, T = 4.38, p_FWE_ = 0.003), but positively to right caudate—dorsal ACC connections ([-9, 47, -2], 56 voxels, T = 4.49, p_FWE_ = 0.003). Faster reactions were related to stronger connectivity between the right IFG and the dorsal ACC ([-6, 44, 7], 183 voxels, T = 5.44, p_FWE_ < 0.001).

For lures, both accuracy and RT were related to the BOLD response of the IFG. The stronger the BOLD response in the IFG, the more accurate were the participants (b = 0.12, SE_b_ = 0.04, t_(609)_ = 3.18, p_FDR_ < 0.01). For the RT, there was an interactive effect with load (b = -0.25, SE_b_ = 0.07, t_(592)_ = -3.36, p_FDR_ < 0.01), participants were slower the stronger the IFG’s BOLD response during the 2-back lures (b = 0.10, SE_b_ = 0.05, t_(273)_ = 2.07, p = 0.04), while faster the stronger the BOLD response during the 3-back condition (b = -0.12, SE_b_ = 0.05, t_(277)_ = -2.42, p = 0.02). Lure performance was not related to BOLD response of any other ROI (all b < 0.07, all t < 1.98, all p_FDR_ > 0.05). Among connectivity parameters, and unlike for targets, higher accuracy was related to stronger connectivity between the frontal ROIs and parietal areas. Specifically, accuracy was positively related to connectivity between the right IFG and the right precentral gyrus ([45, -13, 52], 41 voxels, T = 4.01, p_FWE_ = 0.015) as well as between the right anterior insula and the left SPL ([-30, -52, 46], 36 voxels, T = 4.02, p_FWE_ = 0.027). Faster reactions were related to stronger connectivity between the left IFG and the dorsal ACC ([-9, 32, 25], 33 voxels, T = 3.86, p_FWE_ = 0.036).

## 4 Discussion

The present work revealed differential neural correlates for updating and interference control processes of working memory within the same individuals, using target and lure trials in an n-back task. These findings are particularly relevant in the context of balanced working memory processes, which have been linked to psychological resilience and mental health, and may be especially important for females (Barkley, 2001; McTeague et al., 2016; Shariati et al., 2024). Both updating and interference control showed an overlapping brain network, including the executive fronto-parietal areas, and consistent with the literature, even within our exclusively female sample. Noteworthy, specific patterns arose for each process when considering both activation and voxel-wise connectivity. These patterns included (i) stronger activation and differential connectivity of the MFG and caudate during targets compared with lures, (ii) load-dependent modulation, with cortical activation increasing but subcortical activation decreasing during targets, and the opposite pattern for lures, and (iii) left lateralization for targets, but right lateralization for lures. In the following, these findings are discussed in greater detail.

Regarding (i), activation and connectivity between the bilateral MFG and with parietal areas were increased during targets, while it was decreased during lures. Conversely, during lures, and reflecting interference control, connectivity between MFG and IFG, and between bilateral insulae was stronger. Remarkably, also different subcortical structures showed differential activity and connectivity depending on the process. Specifically, the caudate showed a stronger activation or disinhibition during targets compared with lures, consistent with its role in facilitating “go” type responses versus “stop” trials (Morein-Zamir et al., 2016; O’Reilly & Frank, 2006; Wiecki & Frank, 2013). Although both go and no-go neural populations in the striatum are supposed to be active simultaneously for each trial, the higher activity of the caudate and its increased connectivity to the putamen could reflect the top-down facilitation from the PFC onto striatal structures (O’Reilly & Frank, 2006; Wiecki & Frank, 2013). Importantly, our results corroborate the involvement of these subcortical brain areas not merely as part of the motor control network, but in cognitive processes supporting working memory (Marvel et al., 2019). This was also reflected in the positive relationship between behavioral performance and the caudate activation and connectivity with the anterior cingulate cortex (ACC) during targets. Noteworthy, while ACC is more connected to PFC, it is less connected to striatum (and to a less extent with the insulae) during updating versus interference control.

**Fig. 5.**
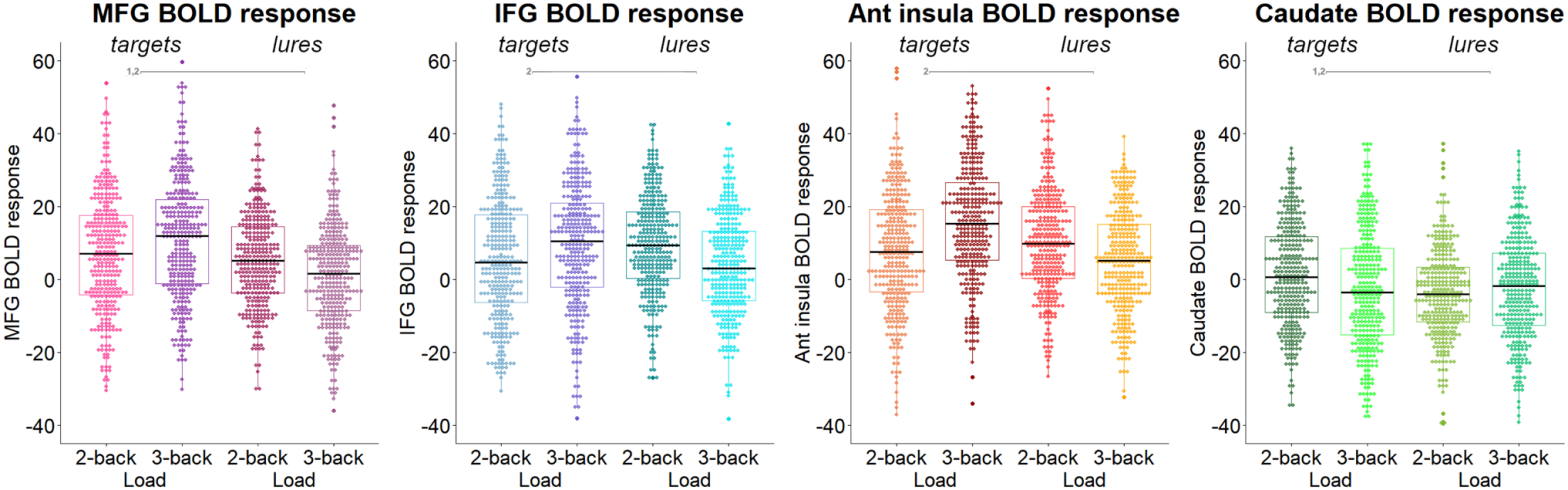
BOLD response of the regions of interest during targets and lures, for each condition and hemisphere. An inverse U-shaped association between increased levels of cognitive load and brain response can be observed for the cortical areas. MFG: middle frontal gyrus; IFG: inferior frontal gyrus; Ant: anterior. 1 indicates trial type significant differences, 2 indicates significant interaction of trial type by load; see Table 2 for specific p-values.

Regarding load-dependent modulation (ii), distinct (and sometimes opposite) patterns emerged for each cognitive process. During updating, cortical ROIs (including MFG, IFG, and anterior insula) showed increased activation with higher load, while the opposite pattern was observed for interference control. In line with prior evidence of an inverse U-shaped relationship between cognitive load and brain response in working memory tasks (Lustig et al., 2009; Manoach, 2003; Nyberg et al., 2009; Reuter-Lorenz & Cappell, 2008), this pattern was observed for 3-back targets compared with 0-back and for 2-back lures compared with 2-back non-lures, when performance decreased and BOLD responses increased. However, during 3-back lure trials, compensatory mechanisms appeared to fail, resulting in both decreased performance and decreased BOLD response (see Fig. 5). Interestingly, and contrary to previous reports, we found an inhibition of the caudate as the load increased during updating (Chang et al., 2007). This “almost” U-shaped function could suggest a potential top-down inhibition at higher demands, possibly mediated by alpha-band activity related to inhibitory control, previously observed during working memory paradigms (Tuladhar et al., 2007). Accordingly, we found the caudate more active in general during the updating versus the interference control process. Extended delay activity under high-load conditions (Sreenivasan & D’Esposito, 2019), and differences in effort-related reward modulation or subjective control (Tricomi et al., 2004), can also not be ruled out.

Load also modulated connectivity, with a crucial role of the IFG and the anterior insula. During updating, both areas increased their activation, and the right IFG became functionally coupled with the left insular cortex/central operculum, while left IFG and right anterior insula decoupled from frontal eye fields and pre/post central gyrus, respectively. These connectivity changes were associated with decreased activation in the decoupled regions, and might reflect a shift from sensorimotor networks toward associative areas (Nee, 2021). During interference control, IFG and anterior insula generally increased their connectivity to each other, reflecting the combined demands of binding and inhibition: lure trials require not only integrating external stimuli with top-down rules, but also suppressing the preponderant response. Relatedly, performance during lure trials depended on IFG function and insula connectivity. These findings corroborate the insula’s pivotal role in reallocating top-down/bottom-up resources, especially with higher binding demands (Goulden et al., 2014; Ko et al., 2013; Menon & Uddin, 2010; Wang et al., 2019), and the engagement of IFG in rule integration and contextual control (Nee, 2021), facilitating adjustments in top-down processing (Craig, 2010). Importantly, these neural patterns may also reflect sex-specific mechanisms. Prior studies have revealed that females recruit prefrontal regions more strongly than males during working memory tasks (Goldstein et al., 2005), with particular engagement of the right IFG (Hill et al., 2014), suggesting potential sex-dependent strategies in working memory processing.

Regarding (iii), an opposite lateralization emerged for updating versus interference control. Specifically for the cortical areas, the left hemisphere was dominant during updating, consistent with the verbal nature of the task (Smith et al., 1998). In fact, stronger connectivity between right MFG and right IFG, areas not dominant for updating, was associated with worsened performance. Conversely, the right hemisphere was dominant for interference control. In general, right IFG and right insula have been linked to conflict detection and interference resolution (Botvinick et al., 2004; Nee et al., 2007), although their precise roles (i.e., cue processing, sustained attention, or response inhibition) remain debated (Dodds et al., 2011; Rodriguez-Nieto et al., 2022). Our results highlight that these regions do not function in isolation; instead, they contribute to various functions within flexible, large-scale brain networks that interact with one another. Thus, the increased activity in the right lateral PFC potentially reflect inhibition (also observed in Rodriguez-Nieto et al. (2022), attentional effort (Wager & Smith, 2003), or/and increased bindings demands during lure trials (Ko et al., 2013; Wang et al., 2019). Connectivity–performance analyses further revealed lateralized patterns. For instance, updating was facilitated by connectivity between the right IFG and the dorsal ACC (dACC), whereas interference control was faster with increased left IFG–dACC connectivity. While top-down bias via the left IFG has been reported to support interference control (Burgess et al., 2011; Jonides & Nee, 2006), IFG–dACC connectivity appears to play a key role for successfully executing both processes.

Even within this sample of young adults, we could see an impact of healthy aging in behavior, activity, and connectivity patterns. As expected, older participants showed slower updating performance and decreased MFG activation (Wang et al., 2019), along with increased MFG–SMG connectivity. While MFG–SMG connectivity did not specifically increase during the updating processes, the recruitment of the SMG may respond to adaptive compensation (Cabeza, 2002; Davis et al., 2008; Reuter-Lorenz & Cappell, 2008). This is in line with the theory of de-differentiation, which suggests that older individuals may rely on broader brain networks than the functionally integrated and specialized network seen in younger individuals (Goh et al., 2011). These findings underscore that age-related variability can modulate working memory-related neural dynamics also in young adult samples.

Finally, this study also poses some limitations that should be acknowledged. While the use of an exclusively female sample represents an important strength for advancing women’s health research and provides a valuable reference for future studies in female populations, it also constrains the generalizability of our findings. Replication in exclusively male samples will be important to assess potential differences. In addition, although our task was designed as a within-subject, mixed block/event-related paradigm (an approach that allows a reliable estimation of the signal with fewer events), the relatively low number of trials per condition remains a limitation that future work should address with longer task designs.

Overall, these findings provide critical insights into the neural dynamics underlying updating and interference control in working memory. Consistent with prior work, fronto-striatal and fronto-parietal connectivity were crucial in adapting to both task demands and individual variability. By directly comparing two trial types in the same task, our results revealed an opposite lateralization and load-dependent modulation based on the predominant process and highlight the nuanced roles of each selected ROIs. IFG and anterior insula (especially the right hemisphere) showed a core binding role under increasing demands. Classically described fronto-striatal areas, such as MFG and caudate, were, on the other hand, most affected by healthy aging. Importantly, these intricate neural dynamics may help explain how balanced working memory supports psychological resilience and mental health, particularly in women during hormonally sensitive periods. Taken together, the present findings might inform theoretical models and provide foundation for future research, advancing our understanding of working memory processes.

## Supplementary Material

Supplementary Material

## Data Availability

Data and scripts used for the statistical analysis in the current manuscript are openly available at https://doi.org/10.17605/OSF.IO/J4CPM. MRI data are available from the corresponding author upon reasonable request.
