# Correction to: N-back task revisited: Comparing the neural correlates of updating and interference control

**DOI:** 10.1162/IMAG.x.1339

**Published:** 2026-08-25

**Authors:** Esmeralda Hidalgo-Lopez, Isabel Noachtar, Belinda Angela Pletzer

**Affiliations:** Department of Psychology & Centre for Cognitive Neuroscience, Paris Lodron University Salzburg, Salzburg, Austria; Department of Psychology, University of Michigan, Ann Arbor, MI, United States; Chronic Pain and Fatigue Research Center, University of Michigan Medical School, Ann Arbor, MI, United States

Unfortunately, there was a coding error caused by a misspelled variable in the script supporting the behavioral and ROI-based analyses in the original article. The error does not affect the significance or direction of the main findings or the interpretation of the results. However, it does affect the numerical values reported in Table 2, as well as some numerical values in the text. Furthermore, some of the brain-behavior associations reported on page 9 no longer survive multiple-comparisons correction.

In the following, we list all corrections in detail. Changes are highlighted in bold font:

## 2.8 Statistical analysis

In the statistical analysis section on page 5 the number of participants excluded from behavioral analyses was incorrectly reported as seven instead of five.

The original sentence:

“A total of **seven** participants for the targets and two participants for the lures were removed from subsequent analyses”

should read:

“A total of **five** participants for the targets and two participants for the lures were removed from subsequent **behavioral** analyses”

## 3.1 Behavioral results

In the behavioral results section on page 6, numerical values in brackets were corrected.

The original sentences:

“Accuracy decreased and RT increased with load (Acc: b = -0.9**3**, SE_b_ = 0.09; RT: b = 0.6**3**, SE_b_ = 0.10) and age (Acc: b = -0.08, SE_b_ = 0.04; RT: b = 0.1**6**, SE_b_ = 0.0**4**). However, the interactive effect between load and trial type indicated that associations with load were stronger for targets than for lures (Acc: b = 0.3**8**, SE_b_ = 0.13; RT: b = -0.**67**, SE_b_ = 0.1**5**; compare Tables 1 and 2).”

should read:

“Accuracy decreased and RT increased with load (Acc: b = -0.9**2**, SE_b _= 0.09; RT: b = 0.6**6**, SE_b_ = 0.10) and age (RT: b = 0.1**5**, SE_b_ = 0.0**5**). However, the interactive effect between load and trial type indicated that associations with load were stronger for targets than for lures (Acc: b = 0.3**7**, SE_b_ = 0.13; RT: b = -0.**70**, SE_b_ = 0.1**4**; compare Tables 1 and 2).”

## 3.2 ROI analyses

In the ROI analyses section on page 6, numerical values in brackets were corrected.

The original section:

“BOLD response was significantly stronger for targets than for lures in the MFG (b = -0.**30**, SE_b_ = 0.**10**) and caudate (b = -0.**29**, SE_b_ = 0.**10**), but not in the IFG (b = -0.1**7**, SE_b_ = 0.**10**) and anterior insula (b = 0.0**5**, SE_b_ = 0.**10**). Irrespective of hemisphere, BOLD response in the cortical ROIs (MFG, IFG, and anterior insula) was positively related to load for targets (all b > 0.**18**, all SE_b_ < 0.0**9**), but negatively for lures (all b < -0.**27**, all SE_b_ **= 0.10**). Conversely, BOLD response of the caudate decreased with load for targets (b = -0.2**4**, SE_b_ = 0.0**7**), but was not significantly related to load for lures (b = 0.0**5**, SE_b_ = 0.**10**). Lateralization was also moderated by trial type for the cortical ROIs. While activation was more left lateralized for targets (MFG: b = -0.1**6**, SE_b_ = 0.05; IFG: b = -0.4**3**, SE_b_ = 0.0**8**; anterior insula: b = -0.20, SE_b_ = 0.0**7**), it was more right lateralized for lures (MFG: b = 0.**20**, SE_b_ = 0.**10**; IFG: b = 0.**40**, SE_b_ = 0.**10**). Age was negatively related to the BOLD response in the MFG (b = -0.13, SE_b_ = 0.04) and caudate (b = -0.11, SE_b_ = 0.04).”

should read:

“BOLD response was significantly stronger for targets than for lures in the MFG (b = -0.**25**, SE_b_ = 0.**08**) and caudate (b = -0.**31**, SE_b_ = 0.**08**), but not in the IFG (b = -0.1**5**, SE_b_ = 0.**09**) and anterior insula (b = 0.0**4**, SE_b_ = 0.**08**). Irrespective of hemisphere, BOLD response in the cortical ROIs (MFG, IFG, and anterior insula) was positively related to load for targets (all b > 0.**24**, all SE_b_ < 0.0**6**), but negatively for lures (all b < -0.**32**, all SE_b_ **< 0.07**). Conversely, BOLD response of the caudate decreased with load for targets (b = -0.2**8**, SE_b_ = 0.0**5**), but was not significantly related to load for lures (b = 0.0**7**, SE_b_ = 0.**06**). Lateralization was also moderated by trial type for the cortical ROIs. While activation was more left lateralized for targets (MFG: b = -0.1**1**, SE_b_ = 0.05; IFG: b = -0.4**0**, SE_b_ = 0.0**6**; anterior insula: b = -0.20, SE_b_ = 0.0**4**), it was more right lateralized for lures (MFG: b = 0.**15**, SE_b_ = 0.**06**; IFG: b = 0.**39**, SE_b_ = 0.**07**). Age was negatively related to the BOLD response in the MFG (b = -0.13, SE_b_ = 0.04).”

Table 2.Numerical values in Table 2 were updated accordingly:

**Table:** The original table:

	Age	Load	Trial Type	Load by Trial Type	Hemisphere	Load by Hemisphere	Trial Type by Hemisphere	Load by Trial Type by Hemisphere
Accuracy	4.63^*^	104.18^***^	107.86^***^	8.79^**^				
RT	12.91^**^	37.12^***^	1.9	20.80^***^				
MFG	11.17^**^	4.49^*^	8.91^*^	10.52^**^	3.11	0.65	6.12^*^	0.83
IFG	2.46	5.73^*^	2.99	16.61^***^	22.57^***^	0.13	34.47^***^	0.15
Ant insula	2.42	31.67^***^	0.29	40.08^***^	4.78	<0.01	3.46~	<0.01
Caudate	6.52^*^	5.87^*^	8.30^*^	4.29^*^	0.28	0.3	0.91	0.18

**Table:** should read:

	Age	Load	Trial Type	Load by Trial Type	Hemisphere	Load by Hemisphere	Trial Type by Hemisphere	Load by Trial Type by Hemisphere
Accuracy	**3.54**	104.49^***^	108.77^***^	8.60^**^				
RT	**9.79** ^**^	**44.83** ^***^	**2.02**	**24.82** ^***^				
MFG	8.96^*^	4.48^*^	9.99^**^	10.**8**2^**^	3.**8**1	0**.9**5	6.93^*^	**1.13**
IFG	**1.73**	**6.26** ^*^	2.**73**	**17.41** ^***^	22.62^***^	0.**09**	34.99^***^	0.1**1**
Ant insula	**1.19**	**30.01** ^***^	0.**13**	**38.51** ^***^	5.26*	<0.01	3.**73~**	<0.01
Caudate	**2.54**	**6.80** ^*^	8.91^**^	**5.14** ^*^	0.**16**	0.**42**	**1.21**	0.**25**

## 3.5 Prediction of Performance

The first sentence in the prediction of performance section on page 9 was corrected to reflect the fact that most associations did not survive multiple-comparisons correction in the updated analyses.

The original sentence:

“For targets, better accuracy and faster reactions were related to higher BOLD response of the MFG (accuracy: b = 0.10, SE_b_ = 0.03, t(614) = 3.10, p_FDR_ < 0.01; RT: b = -0.15, SE_b_ = 0.04, t_(617)_ = -3.89, p_FDR_ < 0.001) and caudate (accuracy: b = 0.12, SE_b_ = 0.03, t_(616)_ = 3.32, p_FDR_ < 0.01; RT: b = -0.10, SE_b_ = 0.04, t_(614)_ = -2.63, p_FDR_ = 0.01), but not the IFG (all b < 0.03 all t < 0.9, all p_FDR_ > 0.05). However, for the anterior insula, a higher BOLD response was related to slower reactions (b = 0.13, SE_b_ = 0.08, t_(615)_ = 3.07, p_FDR_ < 0.01).”

should read:

**“For targets, associations of accuracy to BOLD-response did not survive FDR-correction in any ROI (all |b| < 0.09, all |t| < 2.31, all p_FDR_ > 0.08). For reaction times a significant interaction emerged between BOLD-response in the IFG and load (b = -0.11, SE_b_ = 0.04, t_(480)_ = - 2.54, p_FDR_ = 0.045). This was attributable to significantly faster reactions with higher BOLD-response in the IFG for the 3-back condition (b = -0.09, SE_b_ = 0.04, t_(556)_ = -2.30, p = 0.02), but not for the 2-back condition (b = 0.03, SE_b_ = 0.04, t_(548)_ = 0.64, p = 0.52). No other association between target reaction times and BOLD-response survived FDR-correction.”**

In the corresponding paragraph for lures on page 10, numerical values in brackets were corrected.

The original section:

“The stronger the BOLD response in the IFG, the more accurate were the participants (b = 0.1**2**, SE_b_ = 0.04, t_(_**_609_**_)_ = **3.18**, p_FDR_ **<** 0.01). For the RT, there was an interactive effect with load (b = -0.25,SEb = 0.07, t**_(592)_** = -3.**36**, p_FDR_ < 0.01), participants were slower the stronger the IFG’s BOLD response during the 2-back lures (b = 0.10, SE_b_ = 0.**05**, t**_(273)_** = **2.07**, p = 0.04), while faster the stronger the BOLD response during the 3-back condition (b = -0.12, SE_b_ = 0.05, t**_(277)_** = -2.42, p = 0.02). Lure performance was not related to BOLD response of any other ROI (all b < 0.07, all t < 1.98, all p_FDR_ > 0.05).”

should read:

“The stronger the BOLD response in the IFG, the more accurate were the participants (b = 0.1**1**, SEb = 0.04, t_(_**_597_**_)_ = **2.96**, p_FDR_ **=** 0.01). For the RT, there was an interactive effect with load (b = -0.25, SEb = 0.07, t**_(564)_** = -3.**61**, p_FDR_ < 0.01), participants were **by trend** slower the stronger the IFG’s BOLD response during the 2-back lures (b = 0.**08**, SE_b_ = 0.05, t_(_**_574_**_)_ = **1.65**, p = 0.**10**),while faster the stronger the BOLD response during the 3-back condition (b = -0.17, SEb = 0.05, t**_(586)_** = -**3.25**, p = 0.0**1**). Lure performance was not related to BOLD response of any other ROI (all b < 0.07, all t < 1.**85**, all p_FDR_ > 0.05).”

## Discussion

In the discussion on page 11, the reference to the brain-behavior association that now does not survive multiple comparisons correction needs to be deleted.

The original sentence:

“This was also reflected in the positive relationship between behavioral performance and the caudate **activation and connectivity** with the anterior cingulate cortex (ACC) during targets.”

should read:

“This was also reflected in the positive relationship between behavioral performance and the caudate **connectivity** with the anterior cingulate cortex (ACC) during targets.”

Likewise, in the discussion on page 11, the original sentence:

“Classically described fronto-**striatal** areas, such as MFG and **caudate**, were, on the other hand, most affected by healthy aging.”

should read:

“Classically described fronto-**parietal** areas, such as the MFG and **SMG**, were, on the other hand, most affected by healthy aging.”

**Figure 5:**

Additionally, the figure caption of Figure 5 mentions hemisphere instead of load as a factor.

The original figure caption:

“BOLD response of the regions of interest during targets and lures, for each condition and **hemisphere**”

should read:

“BOLD response of the regions of interest during targets and lures, for each condition and **load**”

