# Supplementary material for: N-back task revisited: Comparing the neural correlates of updating and interference control
Source: Imaging Neurosci (Camb). 2025 Nov 24;3:IMAG.a.1025. doi: 10.1162/IMAG.a.1025 (PMC12645124; doi:10.1162/IMAG.a.1025)
Supplement: Supplementary Material [file IMAG.a.1025_supp.pdf]

SUPPLEMENTARY MATERIAL

Glossary of Brain Region Abbreviations

- **ACgG**: Anterior Cingulate Gyrus
- **AG**: Angular Gyrus
- **AI / AIns**: Anterior Insula
- **amyg**: Amygdala
- **AntPFC**: Anterior Prefrontal Cortex
- **Calc**: Calcarine Cortex
- **Cau**: Caudate Nucleus
- **CO**: Central Operculum
- **FG:** Fusiform Gyrus
- **FO**: Frontal Operculum
- **Hipp**: Hippocampus
- **IOG**: Inferior Occipital Gyrus
- **IFG**: Inferior Frontal Gyrus
- **ITG**: Inferior Temporal Gyrus
- **LOrG**: Lateral Orbital Gyrus
- **MFG**: Middle Frontal Gyrus
- **mSFG**: Medial Superior Frontal Gyrus
- **MTG**: Middle Temporal Gyrus
- **OFuG**: Occipitotemporal Fusiform Gyrus
- **OpIFG**: Opercular Inferior Frontal Gyrus
- **PIns**: Posterior Insula
- **PCu**: Precuneus
- **PO**: Parietal Operculum
- **POrG**: Pars Orbitalis Gyrus
- **PP**: Planum Polare
- **PoCG**: Postcentral Gyrus
- **PreCG**: Precentral Gyrus
- **PT**: Planum Temporale
- **SFG**: Superior Frontal Gyrus
- **SMC**: Supplementary Motor Cortex
- **SMG**: Supramarginal Gyrus
- **SPL**: Superior Parietal Lobule
- **STG**: Superior Temporal Gyrus
- **Th**: Thalamus
- **TrIFG**: Triangular Inferior Frontal Gyrus

Effect of load and age on the voxel-wise BOLD-response in each trial type

Targets. For the targets, the 3-back condition compared to the 2-back enhanced the BOLD-response in frontocingular and insular cortices, while decreased temporoparietal, amygdalae and subcortical areas (Table S3, Fig S1a).

Regarding the age effect, irrespective of the load we observed a negative relation with the activation of the left MFG ([-33, 35, 28], 76 voxels, T = 4.32, p_{FWE} = 0.012), left occipital fusiform and lingual gyrus ([-27, -70, -11], 93 voxels, T = 3.84, p_{FWE} = 0.005), right superior parietal lobe including SMG ([33, -43, 49], 126 voxels, T = 4.19, p_{FWE} = 0.001), and bilateral thalamus ([9, -16, 4], 76 voxels, T = 4.27, p_{FWE} = 0.012), Fig. S2.

Lures: For the lures, the difference in the BOLD-response between lures compared to non-lures was enhanced in pre-postcentral gyri and precuneus during the 3-back compared to the 2-back condition, while decreased in frontocingular, opercular and parietal areas (Table S3, Fig S1b).

No effect of age on the whole-brain BOLD-response was found for the lures vs. non-lures.

a) TARGETS ACTIVATION					b) TARGETS DEACTIVATION				
cluster p_{FWE}	vx	T	x,y,z {mm}	Area	cluster p_{FWE}	vx	T	x,y,z {mm}	Area
0.000	4954	23.71	-30 20 -2	L AIns	0.000	10916	17.24	51 -4 4	R CO
		23.65	-42 17 25	L OpIFG			17.18	-54 -4 1	L PP
		23.21	-6 17 49	L SMC			16.74	-36 2 10	L CO
0.000	3247	20.14	-30 -55 40	L SPL	0.014	71	9.24	-39 29 -17	L POrG
		16.87	-9 -67 55				8.30	-33 35 -14	
0.000	551	11.37	6 -25 -5	R Th	0.001	123	7.14	51 38 1	R TrIFG
		11.31	-6 -16 10	L Th			6.65	39 35 -17	R LOrG
0.004	94	9.31	-48 -58 -11	L ITG	0.000	222	6.50	-27 -94 -2	L IOG
0.032	57	6.15	-48 -49 16	L STG			5.63	-48 -73 4	
0.032	57	5.12	-54 -31 -2	L MTG					

c) LURES ACTIVATION					d) LURES DEACTIVATION				
cluster p_{FWE}	vx	T	x,y,z {mm}	Area	cluster p_{FWE}	vx	T	x,y,z {mm}	Area
0.000	2824	16.09	3 35 46	R mSFG	0.000	4338	13.06	-51 -7 43	L PreCG
		13.63	54 23 19	R OpIFG			10.94	48 -70 -2	R FG
		13.43	45 17 31	R MFG			9.83	-57 2 19	L PreCG
0.000	1016	12.88	-30 20 -8	L AIns	0.000	1400	10.07	9 35 -8	R ACgG
		9.38	-42 14 49	L MFG			8.87	6 47 -5	R mSFG
0.000	165	10.77	-51 -31 -8	L MTG			8.72	18 14 16	R Cau
		6.49	-63 -40 -2	L MTG	0.000	167	9.69	-3 2 58	L SMC
0.000	1037	9.61	42 -64 40	R AG	0.001	121	8.07	36 47 28	R MFG
		9.50	63 -34 -5	R MTG	0.095	37	7.93	-24 32 -14	L POrG
0.000	519	8.63	-51 -55 25	L AG	0.000	230	7.37	27 -19 -20	R Hipp
		8.58	-36 -73 43				7.25	21 -13 -17	
0.000	235	8.31	-9 2 10	L Cau			6.46	30 -31 -8	
0.000	335	7.06	3 -64 37	R PCu	0.000	183	6.65	-36 47 25	L MFG
		6.45	-6 -55 40	L PCu			6.15	-36 38 34	
0.000	136	6.06	-27 -76 -11	L OFuG			6.07	-36 53 13	
		4.70	-9 -91 4	L Calc	0.000	201	6.12	60 5 16	R PreCG
0.023	57	4.66	24 -73 -8	R IOG			4.54	57 -31 16	R PT
		4.33	15 -79 -8	R Calc					

Table S1. Voxel-wise BOLD response during a) 2/3-back targets vs. 0-back targets and b) 0-back targets vs. 2/3-back targets , c) 2/3-back lures vs. 2/3-back non-lures, and d) 2/3-back non-lures vs. 2/3-back lures.

	cluster p_{FWE}	vx	T	x,y,z {mm}	Area		cluster p_{FWE}	vx	T	x,y,z {mm}	Area
TARGETS VS LURES	0.000	2398	16.23	-42 2 37	L PreCG	LURES VS TARGETS	0.000	4661	10.85	39 -4 10	R AIns
			14.20	-6 11 52	L SMC				10.56	39 -4 -8	R PIns
	0.000	2092	15.91	-27 -73 34	L AG				10.40	48 -4 7	R CO
			13.78	-30 -55 49	L SPL		0.000	1661	10.63	-42 -7 13	L CO
	0.000	705	12.72	27 8 55	R MFG				10.47	-54 -4 4	L IFG
			10.90	30 -1 49	R MFG				9.71	-51 -25 16	L PO
	0.000	179	8.43	-48 -61 -11	L ITG		0.000	844	9.29	12 35 52	R SFG
	0.008	78	7.58	30 23 -2	R AI				8.74	-9 35 49	L SFG
	0.006	82	6.24	-9 -19 10	L Th				8.03	9 44 46	R mSFG
			6.12	6 -25 -5	R Th						
	0.013	69	5.04	9 38 -8	R ACgG						
			4.13	6 47 -5	R mSFG						
	0.000	217	4.67	0 -64 13	L PCu						
			4.52	12 -67 7	R Calc						

Table S2. Effect of trial type on the voxel-wise BOLD-response. Areas that showed higher BOLD-response during targets (compared to 0-back) than during lures (compared to non-lures); and lures (compared to non-lures) compared to targets (compared to 0-back).

a)	TARGETS										
	cluster p_{FWE}	vx	T	x,y,z {mm}	Area		cluster p_{FWE}	vx	T	x,y,z {mm}	Area
2-back vs. 3-back	0.000	665	6.95	54 -7 4	R CO	3-back vs. 2-back	0.000	255	9.39	-33 20 -8	L AIns
			6.07	63 -25 13	R PT				4.39	-45 17 4	L FO
			5.47	36 -25 16	R PO		0.000	758	9.38	30 20 -8	R AIns
	0.000	1292	6.47	15 -46 70	R PoCG				6.95	42 23 1	R FO
			5.68	30 -19 70	R PreCG				5.73	30 44 37	R MFG
	0.000	1085	6.27	-57 -4 7	L CO		0.000	734	8.13	0 29 40	L mSFG
			5.99	-54 -1 -2	L PP				6.54	6 32 22	R ACGg
			5.97	-21 -7 -17	L amyg				5.11	-9 32 22	L ACGg
	0.000	339	5.63	24 -4 -17	R amyg		0.000	169	5.94	-51 23 25	L MFG
			5.36	18 23 1	R Cau		0.004	80	5.11	-45 47 4	L MFG
	0.001	102	5.30	-51 -10 46	L PreCG		0.002	98	4.71	42 -58 52	R AG
	0.041	47	4.99	-48 -64 19	L AG		0.001	105	4.54	-30 -76 37	L AG
b)	LURES										
	cluster p_{FWE}	vx	T	x,y,z {mm}	Area		cluster p_{FWE}	vx	T	x,y,z {mm}	Area
2-back vs. 3-back	0.000	164	5.24	-6 38 31	L mSFG	3-back vs. 2-back	0.000	357	5.09	-12 -28 64	L PreCG
			4.72	-3 23 49	L SMC				4.95	0 -25 58	L medPoCG
			4.04	-3 32 40	L mSFG				4.78	-3 -37 55	L medPoCG
	0.000	275	4.81	-45 23 31	L MFG		0.001	115	4.34	9 -52 19	R PCu
			4.73	-42 17 25	L OpIFG				3.77	-9 -58 19	L PCu
			4.57	-45 17 4	L FO						
	0.000	338	4.78	42 20 22	R OpIFG						
			4.63	48 26 34	R MFG						
			4.60	48 32 25							
	0.036	48	3.35	60 -34 1	R MTG						

Table S3. Effect of load on the voxel-wise BOLD-response. a) Areas that showed a load effect during targets (compared to 0-back). b) Areas that showed a load effect during lures (compared to non-lures).

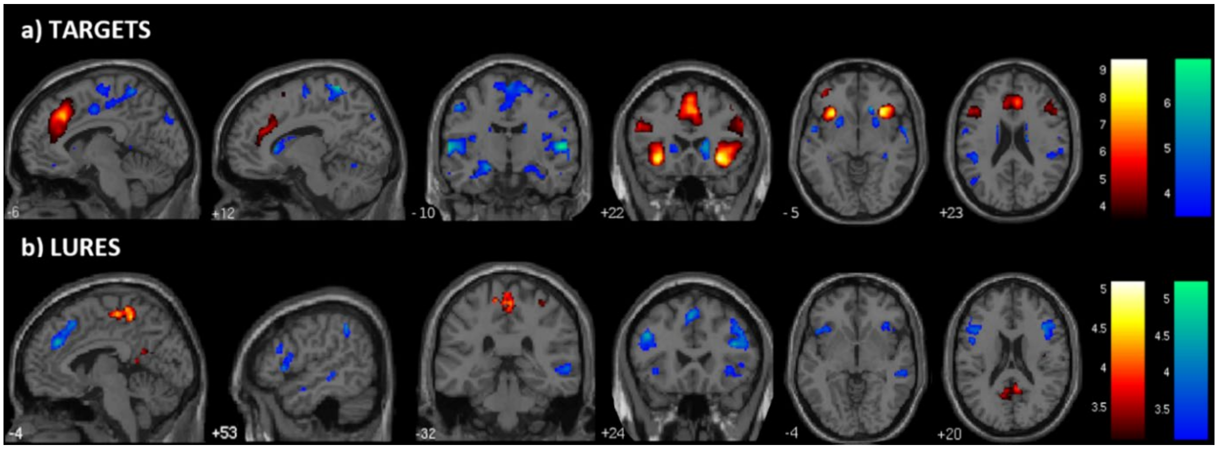

Fig. S1. Effect of load on the voxel-wise BOLD-response. a) Areas that showed a load effect during targets (compared to 0-back). b) Areas that showed a load effect during lures (compared to non-lures). Warm colours indicate positive load effect (3-back vs. 2-back), while cold colours indicate negative load effect (2-back vs. 3-back).

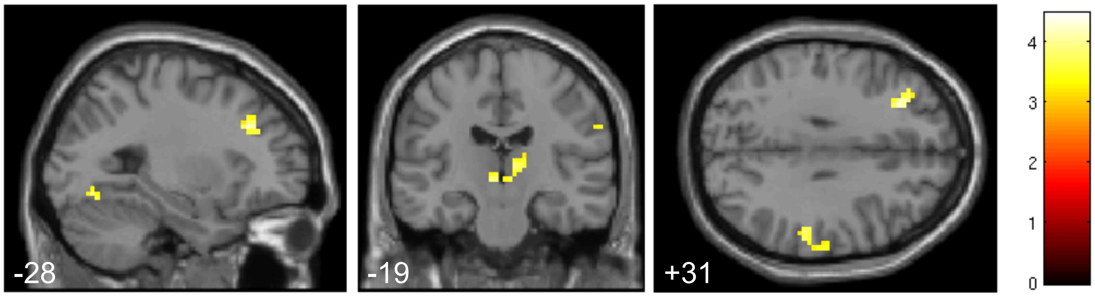

Fig. S2. Effect of age on the voxel-wise BOLD-response. Irrespective of the load we observed a negative relation with the activation of the left MFG, left occipital fusiform and lingual gyrus, right superior parietal lobe, and bilateral thalamus.

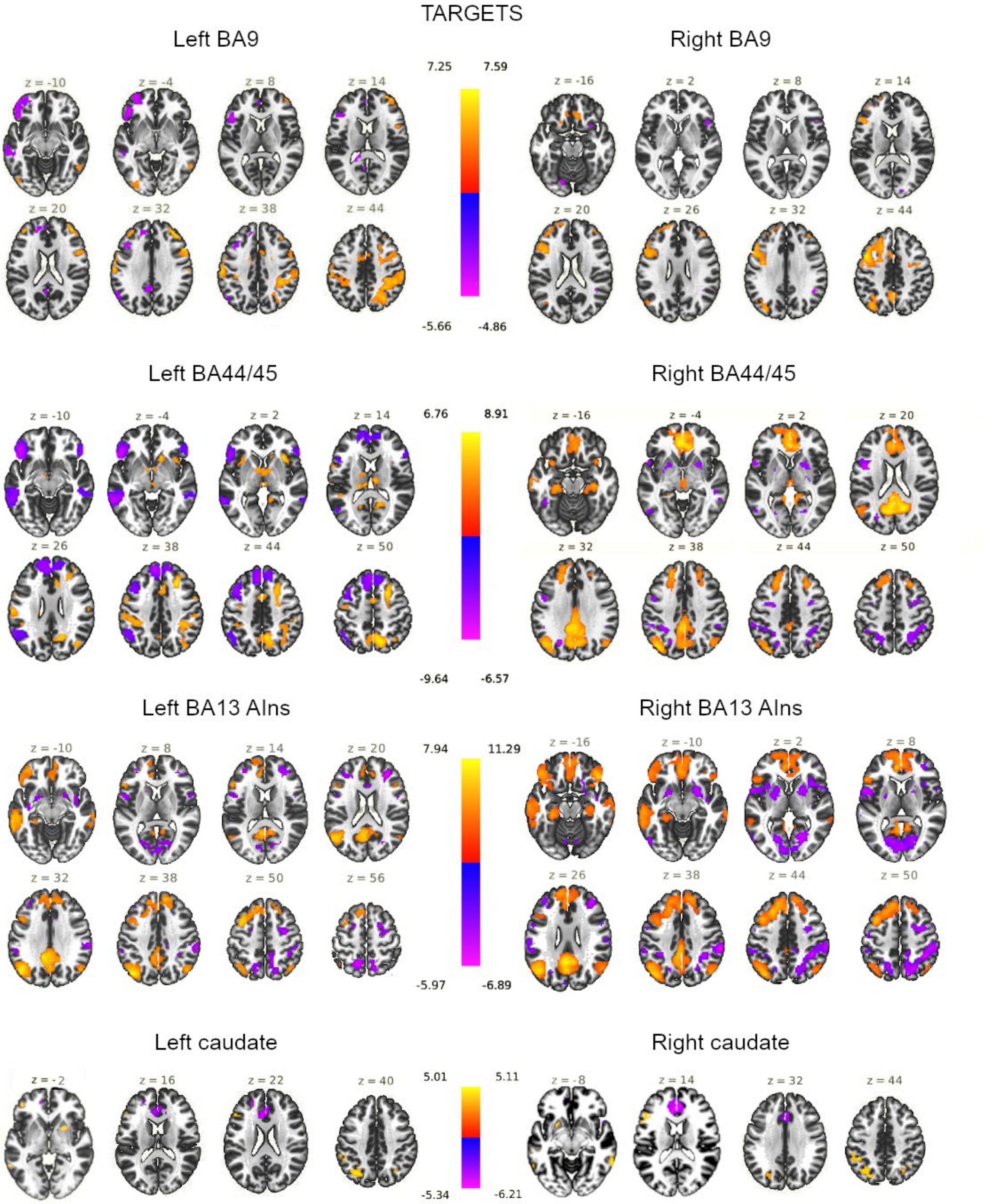

Fig. S3. Connectivity maps for the ROIs during targets irrespective of load.

Voxel-wise connectivity during 2&3-back vs. 0-back targets

During targets, connectivity from the left BA9 increased with bilateral postcentral gyrus, right SPL, right lateral occipital cortex, right MFG, and bilateral supramarginal area, while decreased with the left frontal pole, IFG and MTG. Right BA9 increased its connectivity with the left MFG, superior frontal gyrus, precuneus, left precentral and paracingulate, while decreased with bilateral occipital areas and right AG.

The left BA44/45 increased its connectivity with precuneus, right lateral occipital cortex, right superior and middle frontal gyrus, bilateral pre/postcentral gyrus, left SMG, bilateral insular cortex, bilateral thalamus and anterior and posterior cingulate gyrus. Meanwhile it decreased its connectivity with bilateral frontal pole, superior and inferior Frontal Gyrus, left MFG and middle temporal gyris, and left SMG and AG. Right BA44/45 increased its connectivity with bilateral frontal pole, superior frontal gyri, insular cortex, para/hippocampal areas, anterior/posterior cingulate gyrus, MTG, precuneus, bilateral lateral occipital cortex, left thalamus and MFG. Meanwhile it decreased its connectivity with bilateral putamen, Middle Temporal Gyrus, SMG, SPL, Lateral Occipital Cortex, right MFG and left IFG.

The left AI increased its connectivity with precuneus, posterior cingulate, bilateral Lateral Occipital Cortex, bilateral inferior and Middle Temporal Gyrus, Superior and middle Frontal Gyrus, bilateral frontal pole, bilateral angular gyri, and left para/hippocampal areas. Meanwhile it decreased its connectivity with the right insula, middle and inferior frontal gyri, anterior cingulate, dorsal precuneus, bilateral putamen/pallidum, bilateral SMG, and intracalcarine Cortex. The right AI increased its connectivity with precuneus, anterior and posterior cingulate, bilateral frontal pole, SFG, paracingulate gyri, bilateral inferior and Middle Temporal Gyrus, hippocampi, AG, Lateral Occipital Cortex, and left MFG. Meanwhile it decreased its connectivity with bilateral putamen/pallidum, Precentral Gyrus, Supramarginal Gyrus, SPL, intracalcarine cortex and right MFG.

Both left and right caudate increased its connectivity with bilateral SPL, left Lateral Occipital Cortex, SMG, IFG, MTG, and putamen, while decreased its connectivity with anterior Cingulate Gyrus and bilateral Paracingulate Gyri.

Voxel-wise connectivity during 2&3-back lures vs. 2&3-back non-lures

During lures, and compared to the non-lure trials, the right MFG/BA9 increased its connectivity with bilateral PoCG and right SPL and rAI increased its connectivity with ACC. Conversely, bilateral AI decreased its connectivity with left frontoparietal areas (MFG, IFG, and SMG) and the right caudate decreased its connectivity with left central opercular and insular cortex.

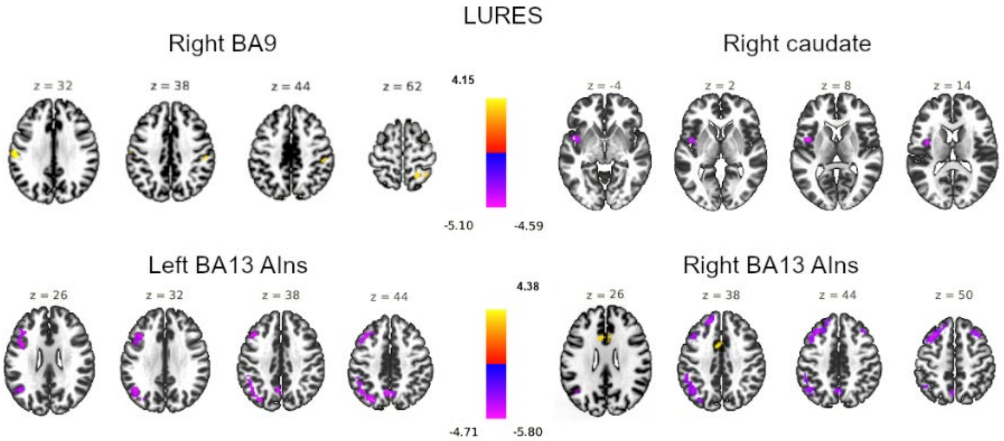

Fig. S4. Connectivity maps for the ROIs during lures irrespective of load.
